# Development and validation of a radiomic prediction model for TACC3 expression and prognosis in non-small cell lung cancer using contrast-enhanced CT imaging

**DOI:** 10.1016/j.tranon.2024.102211

**Published:** 2024-11-27

**Authors:** Weichao Bai, Xinhan Zhao, Qian Ning

**Affiliations:** a Department of Oncology, The First Affiliated Hospital of Xi'an Jiaotong University, Xi'an, Shaanxi Province 710061, China; b Department of Respiratory and Critical Care Medicine, The First Affiliated Hospital of Xi'an Jiaotong University, Xi'an, Shaanxi Province 710061, China

**Keywords:** Contrast-enhanced computed tomography, Radiomics, Transforming acidic coiled-coil protein-3, Non-small cell lung cancer, Clinical prognosis

## Abstract

**Backgrounds:**

Non-small cell lung cancer (NSCLC) prognosis remains poor despite treatment advances, and classical prognostic indicators often fall short in precision medicine. Transforming acidic coiled-coil protein-3 (*TACC3*) has been identified as a critical factor in tumor progression and immune infiltration across cancers, including NSCLC. Predicting *TACC3* expression through radiomic features may provide valuable insights into tumor biology and aid clinical decision-making. However, its predictive value in NSCLC remains unexplored. Therefore, we aimed to construct and validate a radiomic model to predict *TACC3* levels and prognosis in patients with NSCLC.

**Materials and methods:**

Genomic data and contrast-enhanced computed tomography (CT) images were sourced from The Cancer Genome Atlas (TCGA), Gene Expression Omnibus (GEO) database, and The Cancer Imaging Archive (TCIA). A total of 320 cases of lung adenocarcinoma from TCGA and 122 cases of NSCLC from GEO were used for prognostic analysis. Sixty-three cases from TCIA and GEO were included for radiomics feature extraction and model development. The radiomics model was constructed using logistic regression (LR) and support vector machine (SVM) algorithms. We predicted *TACC3* expression and evaluated its correlation with NSCLC prognosis using contrast-enhanced CT-based radiomics.

**Results:**

*TACC3* expression significantly influenced NSCLC prognosis. High TACC3 levels were associated with reduced overall survival, potentially mediated by immune microenvironment and tumor progression regulation. LR and SVM algorithms achieved AUC of 0.719 and 0.724, respectively, which remained at 0.701 and 0.717 after five-fold cross-validation.

**Conclusion:**

Contrast-enhanced CT-based radiomics can non-invasively predict *TACC3* expression and provide valuable prognostic information, contributing to personalized treatment strategies.

## Highlights

• A radiomic model based on contrast-enhanced CT was successfully developed to predict TACC3 expression and NSCLC prognosis.
• High TACC3 expression levels were associated with reduced overall survival compared to lower levels in patients with NSCLC.
• The radiomic model has demonstrated strong predictive accuracy.
• This study highlights the clinical significance of TACC3 as a prognostic biomarker and emphasizes the model's utility in the personalized management of NSCLC.

## Introduction

The incidence rate of lung cancer is the highest in China, where the overall prognosis is suboptimal. Non-small cell lung cancer (NSCLC), a common type of lung cancer, primarily includes adenocarcinoma and squamous cell carcinoma. While surgical resection is the primary treatment, patients with advanced stages exhibit poor prognoses. The treatment focus for patients ineligible for surgery is on extending survival, enhancing quality of life, and achieving long-term survival. Traditional prognostic indicators for NSCLC, including clinicopathological features, serum carcinoembryonic antigen, serum carbohydrate antigen 125, serum squamous cell carcinoma antigen, and computed tomography (CT) findings, are inadequate for the precision required in modern clinical practice. Therefore, investigating novel prognostic biomarkers and prognostic stratification of patients is necessary to identify new markers for personalized precision therapy.

Transforming acidic coiled-coil protein-3 (*TACC3*), a vital member of the TACC family, is a microtubule-associated protein localized to the cell centrosome. *TACC3* forms a complex with chTOG protein and clathrin, stabilizing centrosome microtubules and maintaining centrosome integrity during mitosis. *TACC3* overexpression can facilitate tumor cells in bypassing cell cycle checkpoints by influencing regulatory factors of the cell cycle. As a promoter of epithelial-mesenchymal transition, *TACC3* contributes to the acquisition of migratory and invasive properties in cells, thereby promoting tumor progression [1]. Abnormal Wnt signaling can lead to *TACC3* overexpression; intracellular free β-catenin enters the nucleus, activating oncogenes such as *p53* and *Ras*. Therefore, inhibiting *TACC3* expression in tumor cells also inhibits the p53 signaling pathway and reverses G1 phase arrest in these cells. Qie et al. demonstrated significant *TACC3* upregulation in prostate cancer, where *in vitro* and *in vivo* experiments revealed that *TACC3* knockout inhibited tumor growth [2]. This effect is possibly attributed to the elevated *TACC3* levels disrupting the interaction between filamin A and meckelin, thereby inhibiting the formation of primary cilia in prostate cancer cells. Silencing *TACC3* has been suggested to suppress the Wnt/β-catenin and PI3K/AKT signaling pathways, which are known to regulate cancer stem cell-like characteristics [3]. *TACC3* overexpression is associated with poorer clinical outcomes compared to lower expression levels in various cancers, including breast [4], ovarian [5], lung [6], and gastric [7]. *TACC3* expression is notably upregulated in aggressive tumors, such as centrosome amplification (CA) tumors [4] and metastatic prostate cancer [8]. Recent studies suggest that *TACC3*, especially in its fibroblast growth factor receptor 3 (*FGFR3*)—*TACC3* fusion form, is involved in the progression and resistance to treatment of NSCLC. A study highlighted that *FGFR3-TACC3* fusion occurred in 21 cases (56.8%) of advanced NSCLC patients, indicating its significant prevalence in this cancer subtype [9]. Additionally, overexpression of *TACC3* was correlated with a poor prognosis in lung adenocarcinoma and was associated with immune infiltration, notably of T cells and natural killer cells [10]. Overall, high *TACC3* expression is associated with tumor aggressiveness and poor prognosis across multiple cancer types, highlighting its potential as a prognostic or therapeutic biomarker.

The detection of *TACC3* expression currently relies on methods with inherent limitations. Imaging examinations are routinely conducted in clinical diagnosis. Radiomics is a high-throughput technology that extracts high-dimensional data from conventional medical images to derive numerous image feature parameters [11]. It is non-invasive, enables dynamic monitoring, and quantitatively reflects tumor characteristics [12]. Radiomics technology aids in disease diagnosis, evaluating therapeutic response, and predicting disease progression, recurrence, mortality, and treatment planning [13]. While radiomics technology has been applied to early lung cancer diagnosis and prognostic stratification [14], there is limited research integrating radiomics and molecular biology to predict specific molecular markers like TACC3, which could guide personalized treatment. This study aims to fill this gap by integrating radiomics and molecular biology to enhance personalized treatment strategies.

In this study, we developed and validated a prediction model to non-invasively predict *TACC3* mRNA expression levels in NSCLC using CT-based radiomics technology. The model aims to stratify patients based on their predicted *TACC3* expression levels and identify those with high expression who may benefit from more aggressive treatment regimens. This article follows the Multivariable Prediction Model for Individual Prognosis or Diagnosis (TRIPOD) reporting checklist [15]. A full TRIPOD-compliant report is provided, detailing model development, performance evaluation, and validation procedures. Supplemental Table 1 lists the TRIPOD compliance in detail, ensuring transparency and reproducibility in our approach.

## Methods

### Study design

**Fig. 1:**
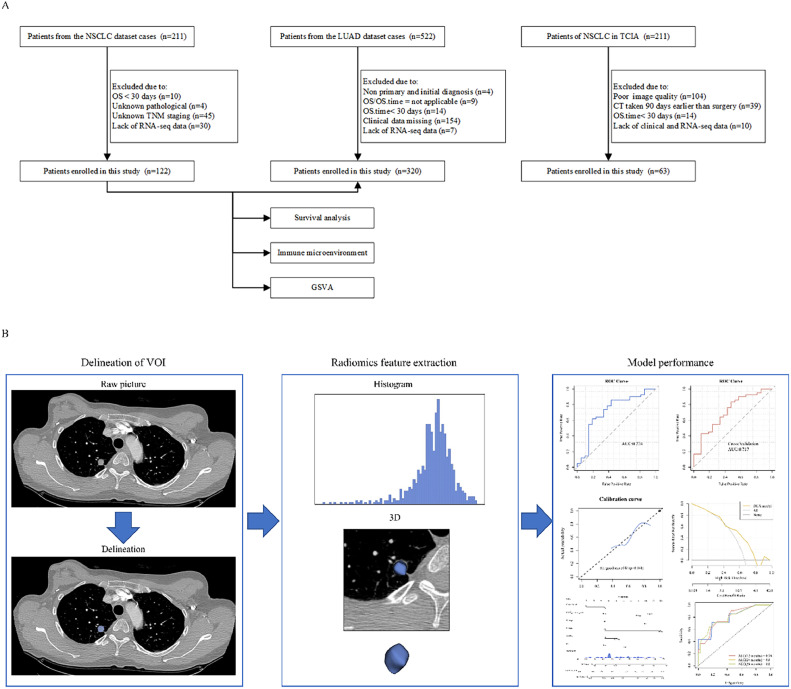
Workflow of this study. A: Sample screening. B: Radiomics analysis.

This exploratory study aimed to investigate and validate the heterogeneity of CT radiomics features in NSCLC and to uncover its underlying prognostic values (Fig. 1).

### Data sources and image acquisition

Clinical, follow-up, and transcriptome sequencing data for NSCLC were obtained from the Gene Expression Omnibus (GEO) database's GSE103584 dataset. Medical imaging data for NSCLC were sourced from the NSCLC Radiogenomics Dataset, which is available at The Cancer Imaging Archive (TCIA). Additionally, clinical, follow-up, and transcriptome sequencing data for lung adenocarcinoma (LUAD) were retrieved from The Cancer Genome Atlas (TCGA) database's TCGA-LUAD dataset (https://portal.gdc.cancer.gov/). A total of 320 cases of LUAD from TCGA and 122 cases of NSCLC from GEO were used for prognostic analysis. Sixty-three cases with both GEO and TICA clinical information were used for radiomics feature extraction, model construction, and evaluation. All samples in this manuscript have been anonymized and are publicly available.

The exclusion criteria were as follows: samples lacking complete clinical data or *TACC3* expression data, samples lacking contrast-enhanced CT images or with poor image quality, samples for which the preoperative CT scan and the surgery had more than 90 days between them, and samples missing clinical or gene expression information (detailed inclusion and exclusion criteria are outlined in Supplemental Table 2). These criteria were defined a priori to reduce potential biases from incomplete or low-quality data, as recommended by TRIPOD. Cutoff values for the analyses were calculated using the R package “survminer.”

### Analysis of group differences

RNA sequencing (RNA-seq) data from TCGA in level 3 HTSeq-FPKM format were processed using the Toil open-source workflow software (https://www.xiantao.love/products) [16]. Log2 transformations were performed to compare *TACC3* expression levels between groups and visualized using the R package “ggplot2.”

### Survival comparisons

Kaplan–Meier survival curves were used to demonstrate variations in survival rates across different groups. Median survival time was defined as the time corresponding to a 50% survival rate. The significance of differences in survival rates between groups was examined using the log-rank test.

### Univariate and multivariate Cox regression analysis

Cox proportional hazards regression models were utilized to assess the impact of multiple variables on survival outcomes. Univariate Cox regression was used for comparative association analysis to analyze the individual association of each variable with OS. Multivariate Cox regression was employed to determine whether specific variables independently influenced OS and explore the combined effects of multiple variables. Hazard ratios (HRs) were calculated, with HRs > 1 signifying increased risk and HRs < 1 indicating reduced risk for OS. Statistical analysis was conducted using the R packages “survival” and “forest plot.”

### Correlation analysis between TACC3 expression and clinical characteristics

Spearman's rank correlation coefficient was used to analyze the correlation between *TACC3* expression and clinical tumor characteristics. Results were visualized using a correlation heat map.

### Enrichment analysis of differentially expressed genes

Gene Set Variation Analysis (GSVA) was used to assess pathway enrichment across samples. The R package “GSVA” was used to calculate the pathway enrichment scores for the Kyoto Encyclopedia of Genes and Genomes (KEGG) and Hallmark gene sets. Differential analysis of the groups with high and low *TACC3* expressions was performed using the R package “limma.” The top 50 pathways were visualized with |t| = 1 as the threshold value. In KEGG pathway analysis, the first 50 out of 186 pathways were visualized, whereas all 50 pathways were displayed in the Hallmark gene set enrichment analysis.

### Correlation between TACC3 expression and immunity-related genes

Spearman's rank correlation coefficient was employed to analyze the correlation between *TACC3* expression and immunity-related genes [17]. Results are presented in a correlation heat map.

### Correlation between TACC3 expression and immune cell infiltration

The gene expression matrix for NSCLC samples was uploaded to the CIBERSORTx database (https://cibersortx.stanford.edu/) to calculate immune cell infiltration for each sample. The R package “corrplot” was used to analyze the correlation between *TACC3* expression and immune cell infiltration levels.

### Radiomics feature extraction and screening

CT images were acquired with a slice thickness of 0.625–3 mm (median: 1.5 mm) using a multi-detector CT scanner. The X-ray tube current ranged from 124 to 699 mA (mean: 220 mA), and the tube voltage ranged from 80 to 140 kVp (mean: 120 kVp). Image acquisition parameters were standardized to ensure consistency across datasets. The subject is positioned supine, with arms naturally resting at their sides, and the scan is completed from the lung apex to the adrenal glands during a single breath-hold [18]. CT images were delineated using the open-source software 3D Slicer. Two experienced radiologists manually delineated the volume of interest (VOI) on images. The radiomic images were resampled to ensure isotropy and minimize variability owing to differences in scanning equipment and protocols and varying lesion sizes among patients. Image normalization was applied to reduce variation in gray scale values of images acquired by different machines.

Radiomics feature extraction standardized 107 features from 63 overlapping samples in GEO and TCIA using the “Pyradiomics” package.

Minimum redundancy maximum relevance (mRMR) was employed to filter the radiomics features. This method considers both the correlation of features with the target variable and the correlation between them. Relevance to the target was assessed through mutual information, averaging the information gained from each feature. Redundancy among features was quantified by summing the mutual information between each pair of features and normalizing by the square of the number of features in the subset. This method ensures that the most informative features are retained while minimizing redundancy, enhancing the model's performance.

Recursive feature elimination (RFE) helped further refine the feature set by ranking the predictive importance of features and systematically removing the least important ones. This iterative involved training the model repeatedly while eliminating less important features and re-evaluating the importance of newly acquired features that contribute maximally to the predictive accuracy and optimal performance of the model. This process was repeated until the optimal feature subset was identified, preventing overfitting and ensuring robustness in the final model.

### Establishment of the radiomics model

Logistic regression (LR), a generalized regression algorithm commonly used for classification problems, is a variation of linear regression. The LR algorithm was applied to the selected radiomics features using the R package “stats” to construct a *TACC3* gene expression predicting model.

The support vector machine (SVM) algorithm, known for constructing high-dimensional hyperplanes to optimize decision boundaries, was employed for modeling the screened radiomics features to predict gene expression. The R package “caret” was used to implement the SVM algorithm.

The DeLong test was used to compare the AUC values before and after cross-validation of the models.

### Evaluation of the radiomics model

The efficacy of the model was evaluated using an internal five-fold cross-validation technique. Evaluation metrics included accuracy, specificity, sensitivity, positive predictive value, and negative predictive value. The receiver operating characteristic (ROC) curve was plotted with the false positive rate (FPR) on the X-axis and the true positive rate (TPR) on the Y-axis. The area under the ROC curve (AUC) indicates the predictive performance of the model, where a larger AUC represents superior model prediction.

In contrast, the precision-recall (PR) curve was plotted with recall, *i.e.*, the TPR on the X-axis and precision on the Y-axis. PR curves were plotted to assess model performance under different class imbalances. The area under the PR curve (PR-AUC) is the mean precision rate calculated for each coverage threshold; a PR curve that bulges toward the upper right corner signifies a more effective model.

To assess the calibration of the radiomics prediction model, a calibration curve was plotted, and the Hosmer–Leme show goodness-of-fit test was performed. The calibration plot provides insights into how well the predicted probabilities match the observed outcomes, a key performance metric in prognostic models as per TRIPOD guidelines.

Additionally, the clinical utility of the radiomics prediction model was evaluated by plotting the decision curve analysis (DCA). This approach quantifies the net benefit of the model and supports the assessment of its clinical applicability.

### Consistency evaluation

The intraclass correlation coefficient (ICC) was used to evaluate the consistency of the extracted radiomics features. This assessment was based on the volume of interest (VOI) delineated by two experienced radiologists. After all cases were delineated by one radiologist, 10 randomly selected samples, identified using the random number table method, were re-delineated by another radiologist. The extracted radiomics features from these VOIs were then compared to assess inter-rater reliability.

### Analysis of inter-group differences in the radiomics model

The radiomics model provided a probability radiomics score (RS) to estimate gene expression levels. The Wilcoxon test was applied to evaluate the association between the RS and *TACC3* expression patterns and identify significant inter-group differences.

### Clinical application

The probability value (RS) derived from the radiomics model was combined with clinical data from 63 patients with NSCLC. We used the PASS (Power and Sample Size) software to evaluate the sample size and the reliability of the results. AUC0 was set to 0.5, as AUC values greater than 0.7 are commonly considered to indicate good model performance [19,20]. AUC1 was set to 0.7, with N+/N- based on RS expression results set at 0.90, and the significance level (α) defined as 0.05 (Supplemental Fig. 1). The analysis revealed that with a sample size of 63, the power of the study was 0.888, indicating robust results (Supplemental Fig. 2). These patients were categorized into high-expression (n = 26) and low-expression (n = 37) groups based on the RS, with cutoff values determined using the R package “survminer.” Stepwise regression screening was performed on the clinical variables to choose those with the minimum Akaike Information Criterion (AIC). A Cox proportional hazards model was then developed to plot a nomogram predicting survival probabilities at 12, 24, and 36 months. Time-dependent ROC curves were generated to evaluate the predictive capacity of various factors over time. The calibration curve was plotted to compare predicted probabilities against actual outcomes, with deviations from the diagonal line indicating prediction errors. DCA was employed to assess the clinical benefits of the predictive model.

### Statistical analysis

Statistical analyses were conducted using R software (https://www.r-project.org/). P < 0.05 was considered statistically significant. Numerical data were presented as mean ± standard deviation, while categorical data were shown as relative distribution frequency and percentage. Baseline characteristics of categorical variables were compared using chi-square tests. Survival comparisons between groups were assessed using Kaplan–Meier curves with log-rank tests. COX proportional hazards regression was used for survival analysis. Spearman's coefficients were calculated to analyze the correlation between RS and other covariates and immunity-related genes.

## Results

### Clinical characteristics of patients with high and low TACC3 expression

A total of 122 patients with NSCLC from the GEO database were included in the survival analysis. These patients were categorized into a *TACC3* high-expression group (n = 74) and low-expression group (n = 48) using a cutoff value of 2.4133. The clinical characteristics of the patients are summarized in Table 1. The histological distribution significantly differed between the *TACC3* high- and low-expression groups.

**Table 1:** Clinical characteristics of patients from the NSCLC dataset cases.

Variables (NSCLC)	Total (n = 122)	Low (n = 48)	High (n = 74)	P
Sex, n (%)				0.06
Female	33 (27)	18 (38)	15 (20)	
Male	89 (73)	30 (62)	59 (80)	
Age (years), n (%)				0.942
< 65	44 (36)	18 (38)	26 (35)	
66 <	78 (64)	30 (62)	48 (65)	
Radiotherapy, n (%)				0.558
No	108 (89)	44 (92)	64 (86)	
Yes	14 (11)	4 (8)	10 (14)	
Chemotherapy, n (%)				0.058
No	86 (70)	39 (81)	47 (64)	
Yes	36 (30)	9 (19)	27 (36)	
Histology, n (%)				0.022
Adenocarcinoma	92 (75)	42 (88)	50 (68)	
Squamous cell carcinoma	30 (25)	6 (12)	24 (32)	
Smoking_status, n (%)				0.566
Nonsmoker	20 (16)	10 (21)	10 (14)	
Current	24 (20)	9 (19)	15 (20)	
Former	78 (64)	29 (60)	49 (66)	
KRAS_mutation_status, n (%)				0.751
Mutant	23 (19)	9 (19)	14 (19)	
Unknown	27 (22)	9 (19)	18 (24)	
Wildtype	72 (59)	30 (62)	42 (57)	
EGFR_mutation_status, n (%)				0.999
Mutant	18 (15)	7 (15)	11 (15)	
Unknown	28 (23)	11 (23)	17 (23)	
Wildtype	76 (62)	30 (62)	46 (62)	
T_stage, n (%)				0.097
Tis/T1	56 (46)	27 (56)	29 (39)	
T2/T3/T4	66 (54)	21 (44)	45 (61)	
N_stage, n (%)				0.877
N0	97 (80)	39 (81)	58 (78)	
N1/N2	25 (20)	9 (19)	16 (22)	
M_stage, n (%)				1
M0	117 (96)	46 (96)	71 (96)	
M1	5 (4)	2 (4)	3 (4)	

EGFR, epidermal growth factor receptor; T, Tumor; M, Metastasis; N, Node.

Additionally, 320 patients with adenocarcinoma from TCGA database were included in the survival analysis and categorized into the *TACC3* high-expression group (n = 168) and low-expression group (n = 152) using a cutoff value of 2.7625. The clinical characteristics of these patients are summarized in Table 2. We ensured that the inclusion/exclusion criteria were uniformly applied to all patient datasets, in line with TRIPOD's emphasis on clearly reporting participant selection methods. We observed significant differences in the distributions of sex, age, radiotherapy, smoking status, and N stage between the *TACC3* high- and low-expression groups.

**Table 2:** Clinical characteristics of patients from lung adenocarcinoma (LUAD) dataset.

Variables (LUAD)	Total (n = 320)	Low (n = 152)	High (n = 168)	P
Sex, n (%)				0.028
Female	172 (54)	92 (61)	80 (48)	
Male	148 (46)	60 (39)	88 (52)	
Age (years), n (%)				0.006
< 65	153 (48)	60 (39)	93 (55)	
66 <	167 (52)	92 (61)	75 (45)	
Radiotherapy, n (%)				0.033
No	288 (90)	143 (94)	145 (86)	
Yes	32 (10)	9 (6)	23 (14)	
Chemotherapy, n (%)				0.133
No	213 (67)	108 (71)	105 (62)	
Yes	107 (33)	44 (29)	63 (38)	
Smoking_status, n (%)				< 0.001
Nonsmoker	45 (14)	29 (19)	16 (10)	
Current	83 (26)	25 (16)	58 (35)	
Former	192 (60)	98 (64)	94 (56)	
Residual_tumor, n (%)				0.935
R0	306 (96)	146 (96)	160 (95)	
R1/R2	14 (4)	6 (4)	8 (5)	
T_stage, n (%)				0.275
T1	108 (34)	58 (38)	50 (30)	
T2	173 (54)	76 (50)	97 (58)	
T3/T4	39 (12)	18 (12)	21 (12)	
N_stage, n (%)				0.004
N0	211 (66)	113 (74)	98 (58)	
N1/N2/N3	109 (34)	39 (26)	70 (42)	
M_stage, n (%)				0.299
M0	228 (71)	113 (74)	115 (68)	
M1/MX	92 (29)	39 (26)	53 (32)	

T, Tumor; M, Metastasis; N, Node.

### Comparison of TACC3 expression levels in normal and tumor tissues

**Fig. 2:**
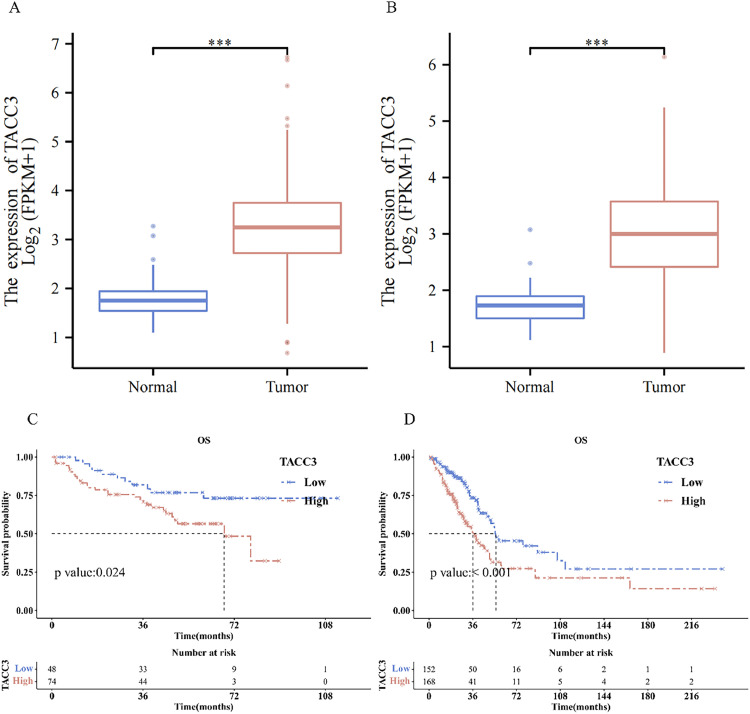
TACC3 expression levels and Kaplan–Meier curve analyses. All tissue samples, both normal and tumor, were obtained from TCGA. The normal tissue samples were derived from paracancerous tissues of cancer patients. The classification of normal and tumor tissues was conducted in strict accordance with TCGA guidelines. A: Expression level of TACC3 in tumor and normal tissues in NSCLC database (P < 0.001). B: Expression level of TACC3 in tumor and normal tissues in LUAD datasets (P < 0.001). C: Kaplan-Meier curves in NSCLC dataset (P=0.024). B: Kaplan-Meier curves in LUAD dataset (P < 0.001). (*P < 0.05; **P < 0.01; ***P < 0.001).

*TACC3* expression levels were significantly increased in tumor tissues compared with those in normal tissues in the NSCLC (P < 0.001) and LUAD (P < 0.001) datasets of the TCGA and GEO database (Fig. 2A and B).

### Comparison of survival data between the high and low TACC3 expression groups

In the NSCLC dataset, the median survival duration for the *TACC3* low-expression group was not achieved owing to the limited number of deaths, whereas it was 68.03 months for the *TACC3* high-expression group (P = 0.024). In the LUAD dataset, the median survival durations were 55.1 and 36.03 months for the *TACC3* low- and high-expression groups, respectively (P < 0.001). Kaplan–Meier curve analyses across datasets indicated that higher *TACC3* expression levels were statistically associated with reduced OS (Fig. 2C and D).

### Associations between TACC3 expression and OS using Cox regression

**Fig. 3:**
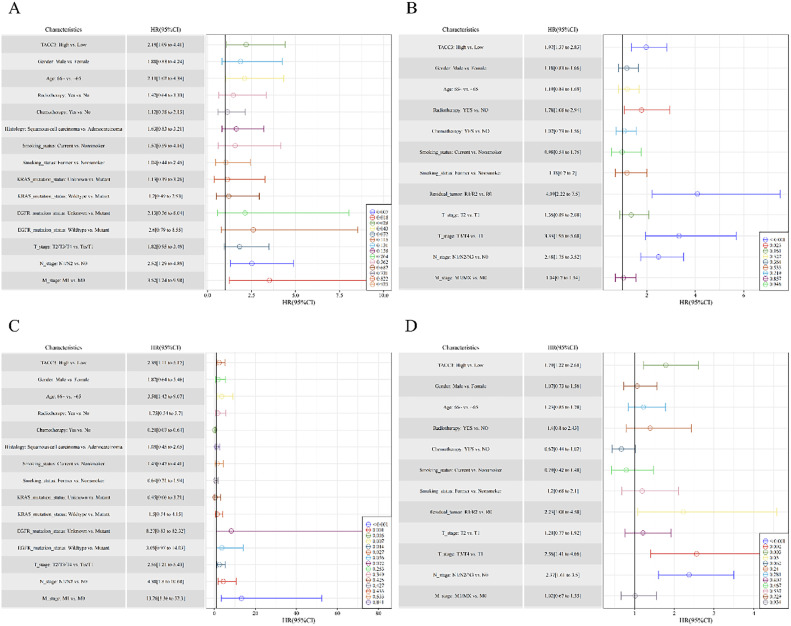
Cox regression analysis. A. Univariate Cox regression analysis in NSCLC database. B. Univariate Cox regression analysis in LUAD database. C. Multivariate Cox regression analysis in NSCLC database. C. Multivariate Cox regression analysis in LUAD database.

Univariate Cox regression analysis helped identify high *TACC3* expression as a risk factor for OS in both the NSCLC (HR = 2.19; 95% CI, 1.09–4.41; P = 0.028) and LUAD (HR = 1.97; 95% CI, 1.37–2.83; P < 0.001) datasets (Fig. 3A and B). Following multivariate adjustment, high *TACC3* expression remained a significant risk factor for OS in the NSCLC (HR = 2.39; 95% CI, 1.11–5.17; P = 0.027) and LUAD (HR=1.79; 95% CI, 1.22–2.61; P = 0.003) datasets (Fig. 3C and D).

### Relationship between TACC3 expression levels and clinicopathological characteristics

**Fig. 4:**
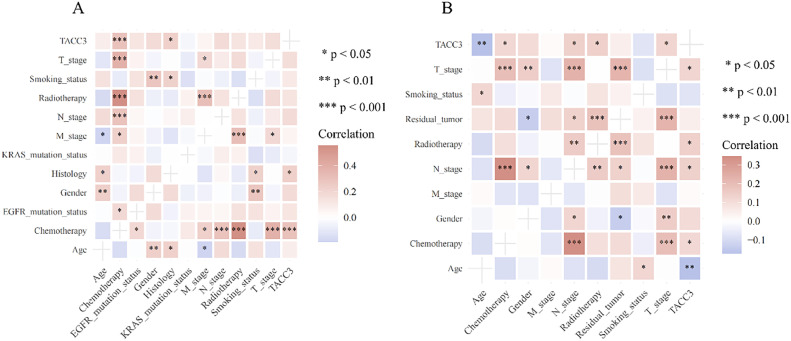
Relationship between TACC3 expression levels and clinicopathological characteristics. A: NSCLC dataset. B: LUAD dataset. (*P < 0.05; **P < 0.01; ***P < 0.001).

A correlation heat map revealed significant positive correlations between *TACC3* expression and chemotherapy and histology in the NSCLC dataset. In the LUAD dataset, *TACC3* expression was significantly positively correlated with chemotherapy, radiotherapy, T stage, and N stage, and significantly negatively correlated with age (Fig. 4A and B).

### GSVA analysis of differentially expressed genes (DEGs) associated with the high and low TACC3 expression groups

**Fig. 5:**
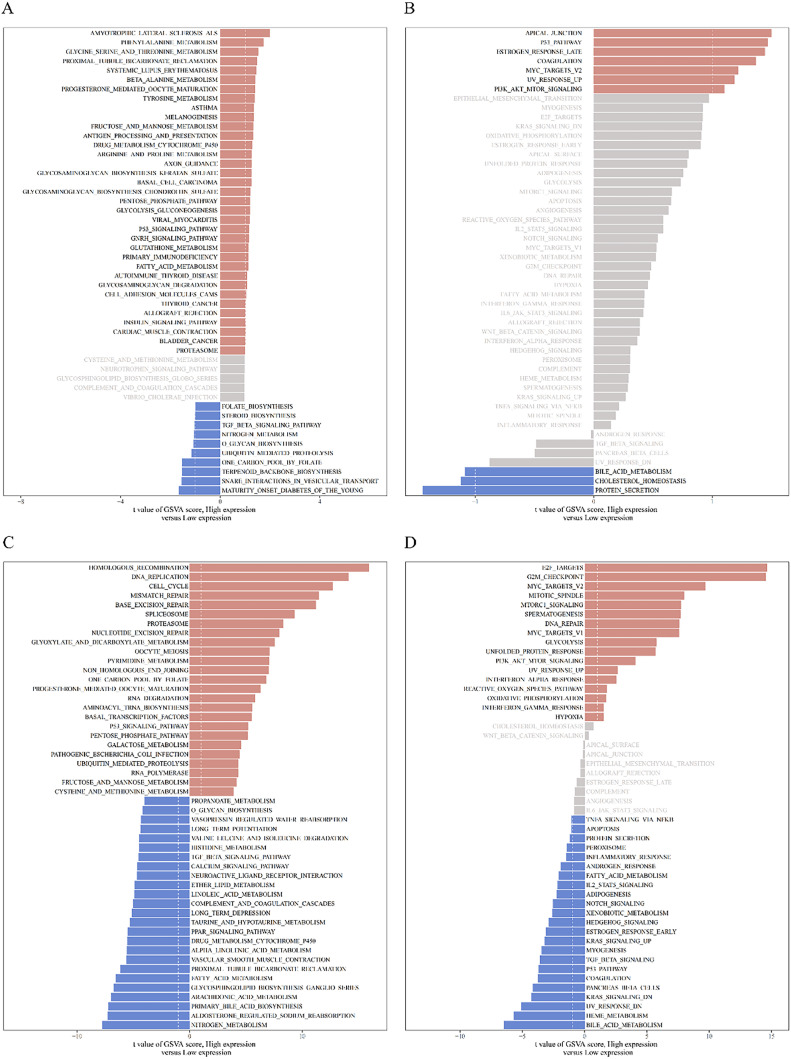
GSVA analysis. A: NSCLC: KEGG gene set. B: NSCLC: Hallmark gene set. C: LUAD: KEGG gene set. D: LUAD: Hallmark gene set.

The analysis of DEGs in *TACC3* high- and low-expression groups revealed significant enrichment in specific pathways associated with *TACC3* expression in NSCLC. Within the KEGG and Hallmark gene sets of the *TACC3* high-expression group, significant enrichment was observed in the p53 signaling pathway (Fig. 5A) and PI3K/AKT/mTOR and p53 pathways (Fig. 5B), respectively. For LUAD, the KEGG gene set analysis indicated significant enrichment in the p53, cell cycle, and mismatch repair signaling pathways within the *TACC3* high-expression group (Fig. 5C). Similarly, in the Hallmark gene set, significant enrichment was found in the DNA repair and PI3K/AKT/mTOR signaling pathways (Fig. 5D).

### Relationship between TACC3 expression levels and immune-related genes and immune cell infiltration

**Fig. 6:**
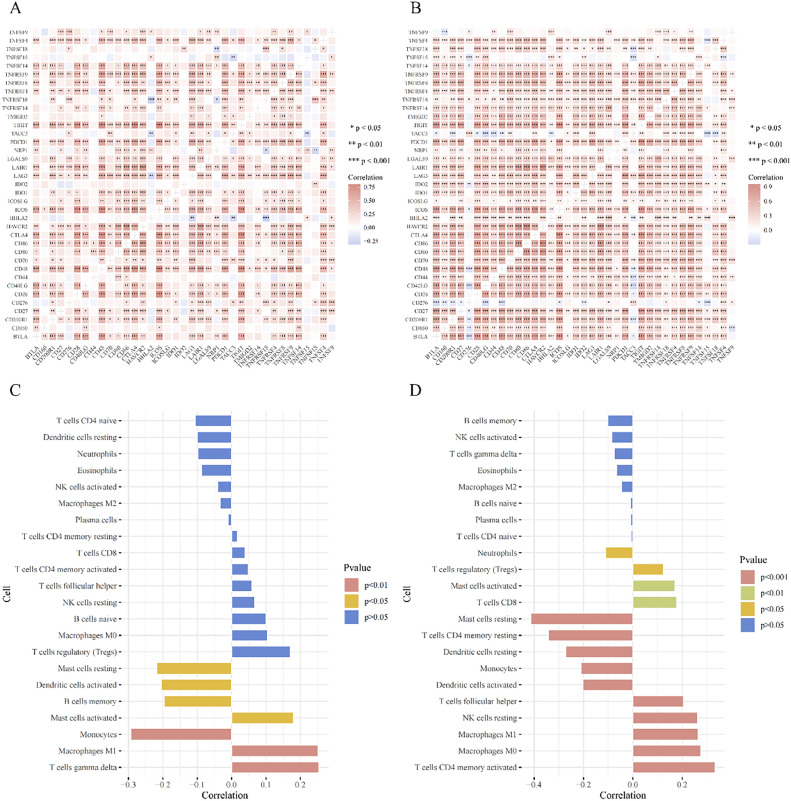
Immune microenvironment. A: NSCLC: Immune-related genes. B: LUAD: Immune-related genes. C: NSCLC: Immune cell infiltration. D: LUAD: Immune cell infiltration.

The correlation heat map analysis indicated significant positive correlations between *TACC3* and immune-related genes such as *CD276, CD70, IDO1, LAG3, PDCD1, TIGIT*, and *TNRSF9* in both the NSCLC and LUAD datasets (P < 0.05; Fig. 6A and B).

Immune cell infiltration analysis indicated significant positive correlations between *TACC3* expression and the relative abundance of M1 Macrophages and activated mast cells (P < 0.05; Fig. 6C and D). In the NSCLC dataset, *TACC3* expression was significantly negatively correlated with activated dendritic cells, monocytes, and memory B cells, while positively correlated with gamma delta T cells. In the LUAD dataset, *TACC3* was negatively correlated with neutrophils, activated dendritic cells, and monocytes, while positively correlated with regulatory T cells, CD8 T cells, follicular helper T cells, M0 Macrophages, and activated memory CD4 T cells (Fig. 6C and D).

### Establishment and evaluation of radiomics models

**Fig. 7:**
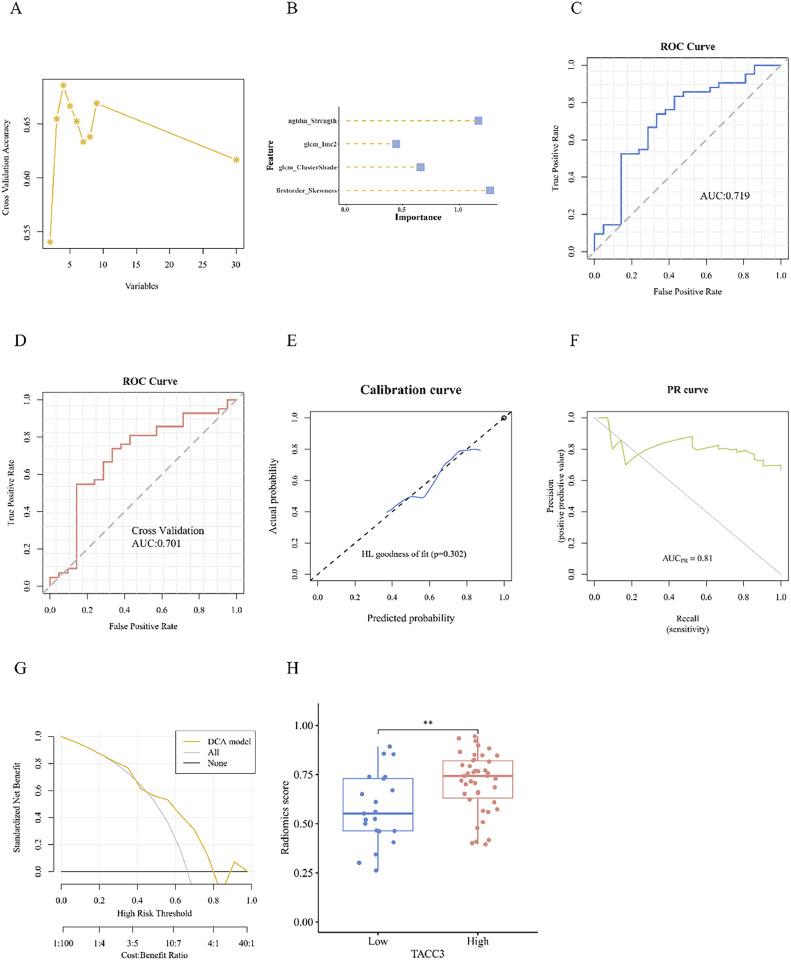
LR model. A: Radiomics features. B: Importance of selected features. C: ROC curve analysis. D: ROC curve analysis with internal 5-fold cross-validation. E: Calibration assessment. F: PR curve. G: DCA. The net benefit is represented on the Y-axis. The yellow curve represents the radiomics model, the gray curve indicates the assumption that all patients were treated, and the straight black line indicates the assumption that no patients were treated. H: Association between RS and *TACC3* expression. (*P < 0.05; **P < 0.01; ***P < 0.001).

We used the mRMR and RFE methods to screen four radiomics features (Fig. 7A). The LR algorithm was used to construct the radiomics model and analyze the importance of the selected features (Fig. 7B). ROC curve analysis revealed an AUC of 0.719 (Fig. 7C). After internal five-fold cross-validation, the AUC was 0.701, demonstrating the robust predictive capability of the model (Fig. 7D). The calibration curve and Hosmer–Lemeshow goodness-of-fit were consistent with the true values (P > 0.05; Fig. 7E). The PR-AUC of the model was 0.81, and the clinical applicability of the DCA was confirmed (Fig. 7F and G). Additionally, the RS distribution was substantially higher in the *TACC3* high-expression group than that in the *TACC3* low-expression group (P < 0.01; Fig. 7H).

**Fig. 8:**
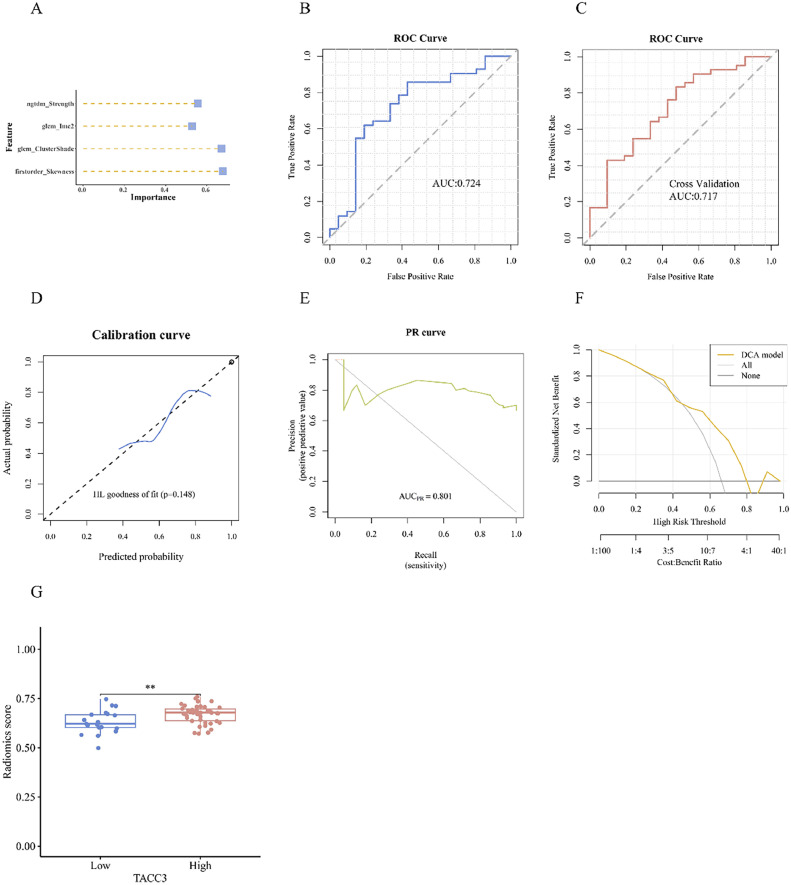
SVM model. A: Importance of selected features. B: ROC curve analysis. C: ROC curve analysis with internal 5-fold cross-validation. D: Calibration assessment. E: PR curve. F: DCA. G: Association between RS and TACC3 expression. (*P < 0.05; **P < 0.01; ***P < 0.001).

To validate the radiomics model, an analysis was performed using the SVM algorithm, specifically examining the significance of selected features (Fig. 8A). ROC curve analysis demonstrated an AUC of 0.724 (Fig. 8B). After internal five-fold cross-validation, the AUC was 0.717, indicating strong predictive power (Fig. 8C), surpassing the AUC value of LR. The calibration curve and Hosmer–Lemeshow goodness-of-fit test demonstrated good agreement between the predicted and actual gene expression levels (P > 0.05; Fig. 8D). The PR-AUC of the model was 0.801, and the clinical applicability of the DCA was confirmed (Fig. 8E and F). RS distribution was significantly different between the high and low gene expression groups, with high RS values in the *TACC3* high-expression group (P < 0.01; Fig. 8G).

The DeLong test revealed no statistically significant difference between the LR and SVM models, with P-values of 0.692 and 0.877 before and after cross-validation, respectively. Given the higher AUC of the SVM model, it was selected for subsequent analyses.

The ICC values for the screened radiomics features all exceeded 0.9, indicating excellent consistency (Table 3).

**Table 3:** Intraclass correlation coefficient (ICC) values of the radiomics model.

Item	Impotence
original_firstorder_Skewness	0.954798681
original_glcm_ClusterShade	0.98119414
original_glcm_Imc2	0.948061244
original_ngtdm_Strength	0.989403167

### Clinical characteristics of the high- and low-RS groups

Significant differences were observed in the sex and histology between the high- and low-RS groups (Table 4).

**Table 4:** Clinical characteristics of the high- and low-radiomics score (RS) groups.

Variables	Total (n = 63)	Low (n = 37)	High (n = 26)	P
Sex, n (%)				0.016
Female	16 (25)	14 (38)	2 (8)	
Male	47 (75)	23 (62)	24 (92)	
Age (years), n (%)				1
< 65	18 (29)	11 (30)	7 (27)	
66 <	45 (71)	26 (70)	19 (73)	
Radiotherapy, n (%)				1
No	57 (90)	33 (89)	24 (92)	
Yes	6 (10)	4 (11)	2 (8)	
Chemotherapy, n (%)				0.392
No	46 (73)	29 (78)	17 (65)	
Yes	17 (27)	8 (22)	9 (35)	
Histology, n (%)				0.004
Adenocarcinoma	49 (78)	34 (92)	15 (58)	
Squamous cell carcinoma	14 (22)	3 (8)	11 (42)	
Smoking_status, n (%)				0.231
Nonsmoker	9 (14)	7 (19)	2 (8)	
Current	18 (29)	8 (22)	10 (38)	
Former	36 (57)	22 (59)	14 (54)	
KRAS_mutation_status, n (%)				0.458
Mutant	12 (19)	8 (22)	4 (15)	
Unknown	10 (16)	4 (11)	6 (23)	
Wildtype	41 (65)	25 (68)	16 (62)	
EGFR_mutation_status, n (%)				0.072
Mutant	13 (21)	11 (30)	2 (8)	
Unknown	10 (16)	4 (11)	6 (23)	
Wildtype	40 (63)	22 (59)	18 (69)	
T_stage, n (%)				0.092
Tis/T1	31 (49)	22 (59)	9 (35)	
T2/T3/T4	32 (51)	15 (41)	17 (65)	
N_stage, n (%)				1
N0	51 (81)	30 (81)	21 (81)	
N1/N2	12 (19)	7 (19)	5 (19)	
M_stage, n (%)				0.564
M0	60 (95)	36 (97)	24 (92)	
M1	3 (5)	1 (3)	2 (8)	
OS, n (%)				0.401
0	39 (62)	25 (68)	14 (54)	
1	24 (38)	12 (32)	12 (46)	
OS. time, Mean ± SD	39.63 ± 22.03	38.04 ± 20.47	41.89 ± 24.31	0.512

EGFR, epidermal growth factor receptor; T, Tumor; M, Metastasis; N, Node; OS, overall survival; SD, Standard Deviation.

### Construction of the predictive nomogram and model evaluation

**Fig. 9:**
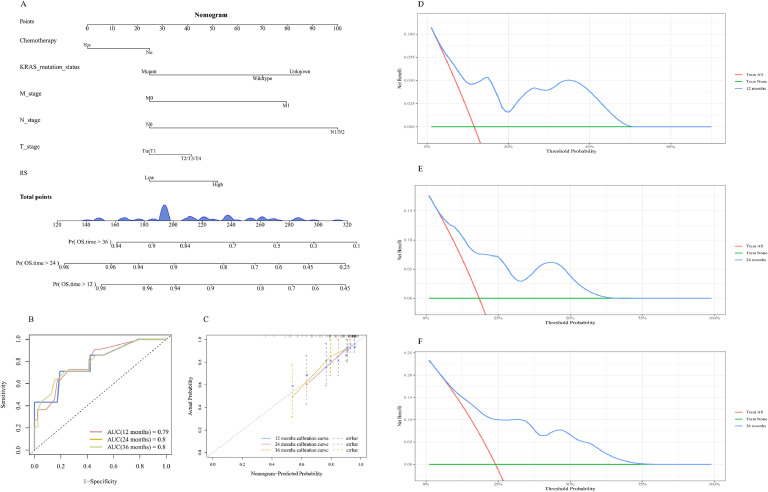
Construction of the predictive nomogram and model evaluation. A: Development of the nomogram for predicting OS. B: Time-dependent ROC for the risk score. C: Calibration curves for the risk score. DCA for 12 months (D), 24 months(E), and 36 months(F).

A predictive model incorporating RS and clinical characteristics such as T stage, N stage, M stage, KRAS mutation status, and chemotherapy was developed using stepwise logistic regression with the minimum Akaike Information Criterion method. Probability values for each patient were labeled on each axis, and the values were summed to obtain a total score (Fig. 9A). The AUC values for the ability of the model to predict OS were 0.79, 0.8, and 0.8 at 12, 24, and 36 months, respectively (displayed in the ROC curves; Fig. 9B). Calibration curves at each time point were close to the diagonal line, indicating minimal prediction error (Fig. 9C). The DCA revealed clinical utility at thresholds ranging between 0.05 to 0.5 at 12 months (Fig. 9D), 0.06 to 0.6 at 24 months (Fig. 9E), and 0.07 to 0.7 at 36 months (Fig. 9F). This predictive nomogram incorporates RS and clinical factors and was validated through AUC, calibration curves, and DCA, consistent with TRIPOD's emphasis on transparent reporting and model performance evaluation.

## Discussion

As tumor therapies advance, traditional prognostic indicators for NSCLC, such as tumor markers and CT, no longer suffice for individualized and precise treatment. The emergence of new biomarkers has introduced the tissue molecular diagnosis concept, which significantly influences therapeutic practices. In this study, we established *TACC3* expression levels as a novel prognostic signature for NSCLC by predicting the correlation between *TACC3* expression and clinical prognosis using non-invasive CT-based radiomics analysis.

*TACC3*, a vital member of the *TACC* family, acts as a multifunctional protein localized to centrosomes during mitosis. *TACC3* participates in the formation and assembly of bipolar spindles [[21], [22], [23]], controlling spindle stability and microtubule nucleation [24,25], and regulating critical oncogenic processes, such as cell proliferation, migration, and invasion. Furthermore, *TACC3* upregulation has prognostic value in solid tumors, including breast [26], lung [6], and prostate cancers [8]. Targeting *TACC3* disrupts spindle formation, induces cell cycle arrest, blocks mitotic progression, and inhibits tumor cell proliferation [4,27]. CA, a hallmark of aggressive cancers [28], involves *TACC3* forming a complex with integrin-linked kinase and chTOG at the centrosomes of CA cancer cells. Inhibiting this complex causes mitotic abnormalities and cell death in CA cancer cells without affecting normal or non-CA cells [29]. *TACC3* plays a critical role in the regulation of several key signaling pathways that are central to cancer progression, including the PI3K/AKT, p53, cell cycle, DNA repair, and mismatch repair pathways. Within the PI3K/AKT signaling pathway, *TACC3* promotes epithelial-mesenchymal transition and cellular proliferation, linking its overexpression to increased tumorigenesis and cell proliferation in various cancers, such as hepatocellular carcinoma and colorectal cancer [1,30,31]. The deletion of *TACC3* induces p53-mediated apoptosis [32], while the loss of p53 upregulates TACC3 expression, rendering cancer cells particularly sensitive to *TACC3* inhibition [4]. *TACC*3′s interaction with the p53 pathway further highlights its role in regulating apoptosis and cellular stress responses, both of which are crucial for maintaining genomic stability. Beyond its involvement in the cell cycle, *TACC3* may also contribute to DNA repair mechanisms, including mismatch repair, which are essential for correcting replication errors and preventing mutations [10]. These findings emphasize the multifaceted role of *TACC3* in cancer biology, underscoring its involvement in multiple critical signaling pathways. The *FGFR3-TACC3* fusion is a common alteration in various cancers [33], including glioblastoma multiforme [34], NSCLC [35], cervical cancer [36], and triple negative breast cancer [37]. *FGFR3-TACC3* was enriched in acquired resistance to epidermal growth factor receptor-targeted therapy in patients with NSCLC [[38], [39], [40]], suggesting that *FGFR3-TACC3* fusion is a recurrent resistance mechanism in NSCLC. In a transgenic murine model expressing *FGFR3-TACC3* with p53 tumor suppressor gene deletion, *FGFR3-TACC3* had an oncogenic function in respiratory epithelial cells [41]. In addition to conventional NSCLC, investigators identified seven cases of lung cancer with clear cell morphology, of which all exhibited the unique *FGFR3-TACC3* fusion site [42]. Among 45 patients with NSCLC resistant to osimertinib, a second targetable alteration was observed in nine patients, including two patients having the *FGFR3-TACC3* mutation [43]. Notably, a patient with epidermal growth factor receptor (EGFR)-mutant advanced NSCLC developed an *FGFR3-TACC3* fusion following osimertinib treatment and exhibited a partial response with a combined therapy of erdafitinib and osimertinib [38]. These observations suggest that dual targeted therapies may provide significant clinical benefits for patients with *FGFR3-TACC3* fusion-related resistance. The *FGFR3-TACC3* fusion is relatively rare, present in only 3.0% of NSCLC samples [44]. However, embracing the idea of personalized medicine implies devoting research efforts to the characterization of tiny subgroups and their possible significance for therapeutic methods. *TACC3* serves as a prognostic factor, justifying investment in diagnostic evaluations in clinical practice. High *TACC3* expression is linked to tumor aggressiveness and poor survival across various cancers, underscoring its potential as a biomarker.

Immune evasion is a key hallmark of cancer, thus the composition of immune cells within the tumor microenvironment plays pivotal roles in determining tumor progression, metastasis, and patient outcomes. The immune microenvironment characterized by the infiltration of cytotoxic T cells, particularly CD8+ T cells, is often associated with improved prognosis and enhanced response to immunotherapy [45]. Conversely, an immune-suppressive environment driven by regulatory T cells, myeloid-derived suppressor cells, and immunosuppressive cytokines can promote tumor growth and metastasis [46]. Chen et al. discovered that the *TACC3* expression in patients with LUAD correlates with the infiltration of various tumor-infiltrating immune cells [10]. Similarly, we demonstrated that *TACC3* expression correlates with the infiltration of different immune cell types, potentially influencing patient prognosis through the immune microenvironment. Furthermore, correlation analysis with immune genes revealed a positive correlation between *TACC3* and several immune checkpoint genes, suggesting coordinated expression of these genes in various signaling pathways, thereby impacting tumor response to immunotherapy.

Given the poor prognosis of NSCLC, especially in patients with intermediate and advanced stages who are ineligible for surgery, prognostic stratification is critical for developing precise treatment strategies to prolong survival. Machine learning studies draw patterns from data to solve tasks and make accurate predictions. Radiomics has become increasingly relevant in predicting disease prognosis [47], with image-based machine learning applications gaining traction in clinical oncology. An effective prediction of mortality risk was achieved using a multi-layer perceptron model combining clinical features, *EGFR* status, and radiomics. This model may facilitate individualized management of patients with lung cancer and brain metastases [48]. However, Ge et al [49]. extracted radiomics features from lung nodules and incorporated different feature selection methods, and only 16 of 2100 tested combinations had an AUC > 0.65, suggesting that there is no clear pathway to obtain reliable CT NSCLC radiomics.

*TACC3* expression is associated with poor prognosis in lung cancer [10]. Our Kaplan–Meier curve analysis in both NSCLC and LUAD datasets confirmed that high *TACC3* expression is associated with reduced OS. Thus, non-invasive prediction of *TACC3* expression using radiomics enhances clinical prognosis accuracy. In this study, we used the mRMR and RFE algorithms to select the optimal feature set and construct the LR and SVM models predicting *TACC3* expression. The prediction models achieved AUC of 0.719 and 0.724, respectively, which remained consistent after five-fold internal cross-validation. The calibration curve and Hosmer–Lemeshow goodness-of-fit test indicated a strong agreement between the predicted and actual gene expression probabilities, whereas the DCA demonstrated the high clinical utility of the models. Our findings revealed a positive correlation between RS for gene expression levels and *TACC3* expression. Furthermore, integrating RS with clinical features enabled us to create nomograms predicting 12-, 24-, and 36-month survival probabilities, with model evaluations suggesting minimal prediction error. This indicates that our radiomics-based model effectively predicts *TACC3* expression and may significantly assist in clinical prognostic stratification, offering both stability and strong diagnostic performance. Compared to other radiomics models developed for NSCLC prognosis, our model demonstrates competitive performance. For example, Similarly, Wu et al. reported an AUC value of 0.75 for the validation cohort of patients with stage IB-IV NSCLC responding to immunotherapy [50]. Similarly, Li et al. developed a radiomics model predicting prognosis in stage IV ALK-positive NSCLC, with a concordance index of 0.717 and an AUC of 0.824 in their validation cohort [51].

The predictive capability of the radiomics model for *TACC3* expression shows significant potential. In patients predicted to have high *TACC3* expression, this may indicate the need for more aggressive treatments, such as combination therapies or immunotherapy, given its association with immune cell infiltration. In cases with *FGFR3-TACC3* fusion mutations, *FGFR* inhibitors like erdafitinib could offer an effective therapeutic option. Integrating the *TACC3* predictive model into diagnostic workflows could assist in identifying patients suited for targeted therapies. Additionally, the model could facilitate treatment monitoring by enabling adjustments based on dynamic changes in *TACC3* expression. In conclusion, *TACC3* serves as an independent prognostic biomarker, identifying high-risk patients, guiding more intensive therapeutic interventions, and correlating with tumor immune infiltration, thereby informing the selection of targeted therapies and supporting personalized, precise treatment strategies [10,52].

However, this study has certain limitations. First, the inherent variability in CT data and image quality sourced from public databases may affect predictive analysis results. Contrast-enhanced CT increases the density contrast between lesions and adjacent normal tissues through contrast agent injection, producing non-quantitative, potentially subjective results. We addressed this by having two experienced radiologists manually outline the VOI and evaluate the consistency of extracted radiomics features using the ICC. Future studies could improve consistency by using advanced imaging techniques like dual-energy CT or positron emission tomography-CT. Second, this retrospective study, which utilized data from a public database, may introduce selection bias. To mitigate this limitation, future studies should incorporate prospective designs with longer follow-up periods and more comprehensive clinical data. Integrating multi-omics (genomics, transcriptomics, proteomics, and metabolomics) with radiomics could further clarify *TACC3*'s role in NSCLC. Finally, the sample size of our study was relatively small. Future research should prioritize incorporating larger, more diverse populations to validate the model in independent cohorts and assess its generalizability through multi-center clinical trials. Integrating *TACC3*-based radiomics with other biomarkers, like immune checkpoint inhibitors or *EGFR* mutations, could enhance patient stratification and personalization, expanding the model's clinical utility.

## Conclusion

Our results revealed a significant correlation between *TACC3* expression and the prognosis of patients with NSCLC. We developed two radiomics models based on the characteristics of contrast-enhanced CT radiomics that can effectively predict *TACC3* expression signatures. Our models exhibited favorable predictive efficiency and have the potential to be a prognostic tool for patients with NSCLC with broad clinical applications. The potential for the models to be integrated into clinical workflows to improve patient stratification and treatment planning was considered. Further validation in large, diverse cohorts is critical, as well as the exploration of the model's applicability to other cancer types or its use in guiding personalized treatment.

## Data availability statement

In this study, publicly available datasets were evaluated. Clinical, follow-up, and transcriptome sequencing data were obtained from the Gene Expression Omnibus (GEO, https://www.ncbi.nlm.nih.gov/geo/) database's GSE103584 dataset and The Cancer Genome Atlas (TCGA, https://portal.gdc.cancer.Gov/) database's TCGA-LUAD dataset. Medical imaging data were sourced from The Cancer Imaging Archive (TCIA, https://www.cancerimagingarchive.net/).

## Funding

This study was supported by the 10.13039/501100013064Key Research and Development Program of Shaanxi (Program No.2022SF-141).

## CRediT authorship contribution statement

**Weichao Bai:** Writing – review & editing, Writing – original draft, Methodology, Investigation, Formal analysis. **Xinhan Zhao:** Writing – review & editing, Formal analysis, Conceptualization. **Qian Ning:** Writing – review & editing, Writing – original draft, Supervision, Investigation, Funding acquisition, Conceptualization.

## Declaration of competing interest

The authors declare that the research was conducted in the absence of any commercial or financial relationships that could be construed as a potential conflict of interest.

## Appendix Supplementary materials

Image, image 1

Image, image 2

Image, application 3

Image, application 4
