# Supplementary figures and images for: Development and validation of a radiomic prediction model for TACC3 expression and prognosis in non-small cell lung cancer using contrast-enhanced CT imaging
Source: Transl Oncol. 2024 Nov 27;51:102211. doi: 10.1016/j.tranon.2024.102211 (PMC11635781; doi:10.1016/j.tranon.2024.102211)

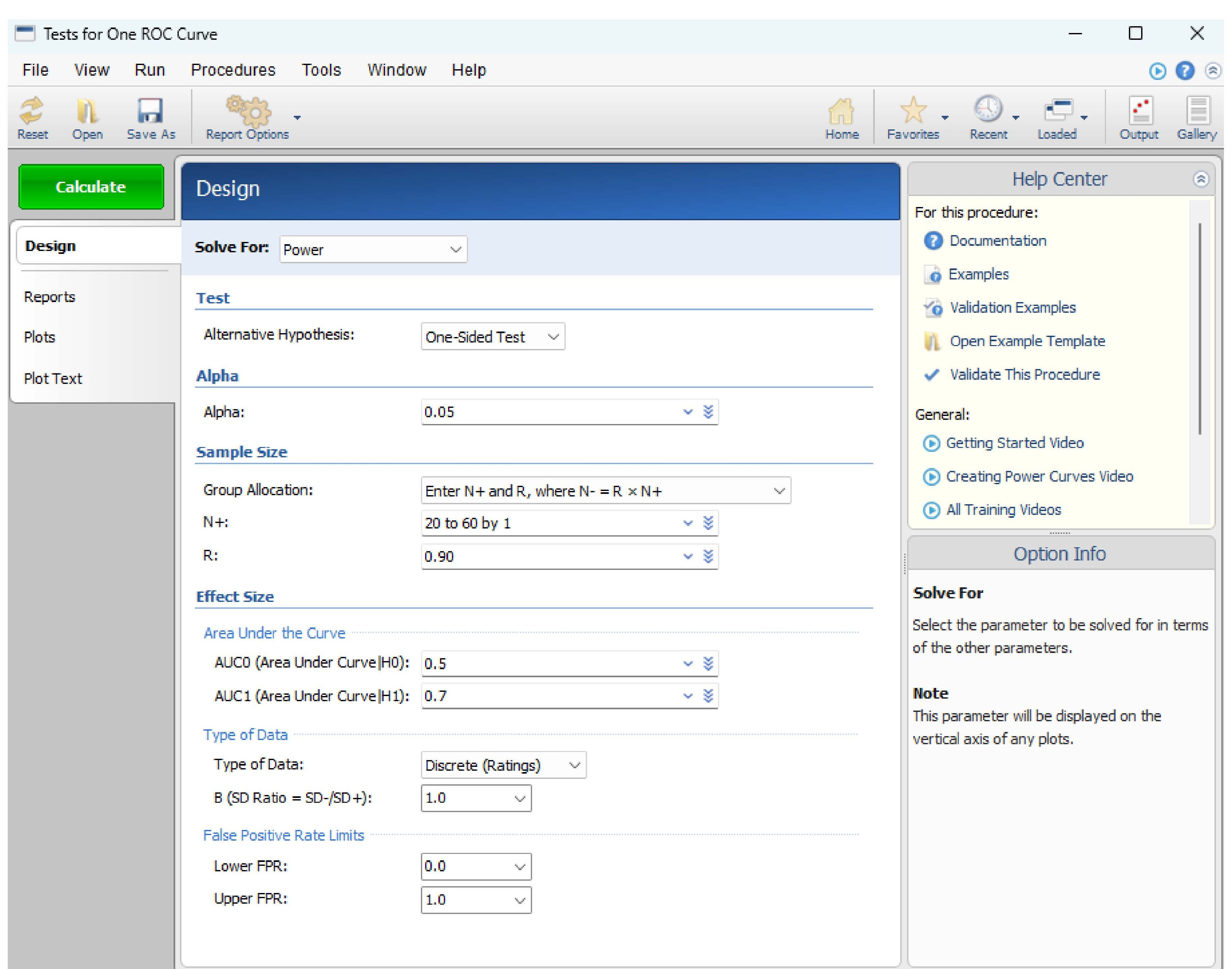

Supplement: Supplementary file 1 [file mmc1.jpg]

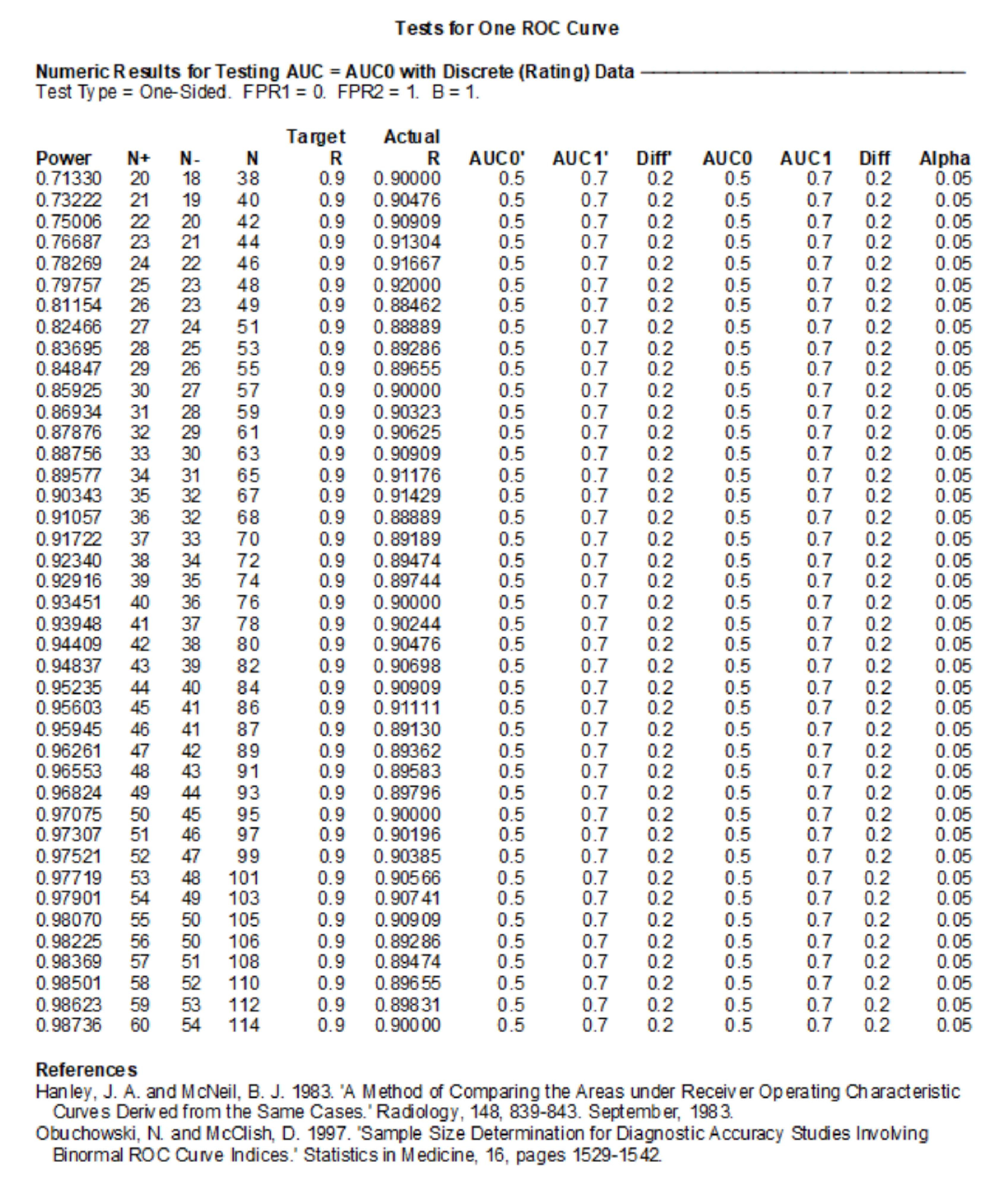

Supplement: Supplementary file 2 [file mmc2.jpg]
