# Supplementary material for: Development and validation of a radiomic prediction model for TACC3 expression and prognosis in non-small cell lung cancer using contrast-enhanced CT imaging
Source: Transl Oncol. 2024 Nov 27;51:102211. doi: 10.1016/j.tranon.2024.102211 (PMC11635781; doi:10.1016/j.tranon.2024.102211)
Supplement: Supplementary file 4 [file mmc4.docx]

Supplemental Table 2: Inclusion and exclusion criteria for study sample selection.

| Description | Excluded samples | Remaining samples |
| --- | --- | --- |
| NSCLC radiogenomics - Clinical data |  |  |
| Total samples | - | 211 |
| Excluded: OS < 30 days | 10 | 201 |
| Excluded: Unknown pathological classification | 4 | 197 |
| Excluded: Unknown TNM staging | 45 | 152 |
| Screening: RNA-seq available | 30 | 122 |
| NSCLC radiogenomics - CT |  |  |
| Total samples | - | 211 |
| Excluded: Poor quality | 104 | 107 |
| Excluded: CT taken 90 days earlier than surgery | 39 | 73 |
| Intersection of clinical data and RNA-seq data | 10 | 63 |
| TCGA-LUAD* |  |  |
| Total samples | - | 522 |
| Screening: Primary and initial diagnosis | 4 | 518 |
| Excluded: OS/OS.time = not applicable | 9 | 509 |
| Excluded: OS.time < 30 days | 14 | 495 |
| Excluded: Clinical data missing | 154 | 327 |
| Screening: RNA-seq available | 7 | 320 |

CT, computed tomography; GEO, Gene Expression Omnibus; LUAD, lung adenocarcinoma; NSCLC, non-small cell lung cancer; OS, overall survival; TCGA, The Cancer Genome Atlas.
